# Complete mitochondrial genome of the euryhaline monogonont rotifer *Brachionus paranguensis* (Rotifera, Brachionidae)

**DOI:** 10.1080/23802359.2019.1704655

**Published:** 2020-01-10

**Authors:** Beom-Soon Choi, Duck-Hyun Kim, Jin-Sol Lee, Hee-Jin Kim, Atsushi Hagiwara, Jae-Seong Lee

**Affiliations:** a Phyzen Genomics Institute, Seongnam, South Korea;; b Department of Biological Science, College of Science, Sungkyunkwan University, Suwon, South Korea;; c Institute of Integrated Science and Technology, Nagasaki University, Nagasaki, Japan;; d Organization for Marine Science and Technology, Nagasaki University, Nagasaki, Japan

**Keywords:** Monogonont rotifer, complete mitochondrial genome, *Brachionus paranguensis*

## Abstract

The two complete mitochondrial genomes were sequenced from the euryhaline monogonont rotifer *Brachionus paranguensis*. The mitochondrial genome sequences were 11,603 bp and 12,901 bp in size, and the gene order of 12 protein-coding genes (PCGs) were identical to those of the marine rotifer *Brachionus plicatilis*, but the positions of some tRNAs (e.g. tRNA-Ile, tRNA-Leu[TTA], tRNA-Phe, and tRNA-Leu[CTC]) of mitochondrial DNA I were different between *B. paranguensis* and *B. plicatilis* mitochondrial genomes. Of 12 PCGs, four genes (*ND1*, *ATPase 6*, *ND5*, and *ND3*) had incomplete stop codons. Furthermore, the start codon of *ND4*, *ND5*, and *CO3* genes was ATT, while the start codon of other PCGs was ATG. The base composition of 12 PCGs in *B. paranguensis* mitochondrial genomes was 26.6% for A, 43.0% for T, 17.65% for C, and 12.75% for G, respectively.

To date, in the euryhaline rotifer *Brachionus* spp., only a few mitogenomes were reported, such as *Brachionus plicatilis* (Suga et al. 2008), *Brachionus koreanus* (Hwang et al. 2013; Hwang et al. 2014), and *Brachionus rotundiformis* (Kim et al. 2017), while complete mitogenomes of the freshwater rotifers *Brachionus calyciflorus* (Nie et al. 2016; Choi, Lee, Hagiwara, et al. 2019), *Brachionus rubens* China (GenBank KJ489417 and KJ489418), and *B. rubens* Japan (Choi, Lee, Lee, et al. 2020) have been published. The L morphotypes of the euryhaline *Brachionus* rotifer consist of four groups; L1 for *B. plicatilis*, L2 for *Brachionus manjavacas*, L3 for *Brachionus asplanchnoidis*, and L4 for *B. paranguensis*. However, to date, there is no report on complete mitochondrial genome for *B. paranguensis*, while the description of this new species has been reported with DNA taxonomy, morphology, and ecology (Guerrero-Jiménez et al. 2019). The analysis of the complete mitochondrial genome in the rotifer *B. paranguensis* is important to study the population genetics with identification of field-sampled and laboratory strains, as there are many cryptic species in the euryhaline rotifer *B. plicatilis* species complex (Mills et al. 2017). In this study, we identified two complete mitochondrial genomes of the euryhaline monogonont rotifer *B. paranguensis* to better understand the phylogenetic placement of *B. paranguensis* in the rotifer *Brachionus* clades.

The adults *B. paranguensis* were collected by netting from Little Fish Lake, Nevada, in the USA (38°34′58.8″N, 116°28′25.2″W) in October 1994 by Prof. Stephanie Hampton and maintained at the Laboratory of Prof. Atsushi Hagiwara, Nagasaki University in Japan through the donation of Prof. Terry Snell (Georgia Institute of Technology, Atlanta, GA, USA). The type specimen was deposited in the ichthyological collection of the Faculty of Fisheries, Nagasaki University (FFNU) under the accession no. FFNU-Rot-0002. We sequenced genomic DNA of *B. paranguensis* using PacBio long-read sequencing (Pacific Biosciences, Menlo Park, CA) and assembled the long sequence reads with Flye assembler v2.4.2 (https://github.com/fenderglass/Flye). The initial assembly consisted of 1,800 contigs, which were polished, using Pilon V1.23 (https://github.com/broadinstitute/pilon/releases/), with the filtered sequences (19,484,112,568 bp) generated from 500 bp Paired-End library sequencing on the Illumina HiSeq 2500 platform (GenomeAnalyzer, Illumina, San Diego, CA) and obtained two complete mitochondrial DNAs.

The complete mitochondrial genomes of *B. paranguensis* were 11,603 bp (mitochondrial DNA I; GenBank no. MN755861) and 12,901 bp (mitochondrial DNA II; GenBank no. MN755862) in size. The direction and placement of 12 protein-coding genes (PCGs) of *B. paranguensis* were identical to those of *B. plicatilis* of the morphotype L4 of the rotifer *B. plicatilis* species complex (Suga et al. 2008). Of 12 PCGs, four genes (*ND1*, *ATPase 6*, *ND5*, and *ND3*) had incomplete stop codons. Furthermore, the start codon of *ND4*, *ND5*, and *CO3* genes was ATT, while the start codon of other PCGs was ATG. The base composition of 12 PCGs in *B. paranguensis* mitogenome revealed 26.6% for A, 43.0% for T, 17.65% for C, and 12.75% for G, respectively. The mitochondrial genome A + T base composition (69.6%) of 12 PCGs was higher than G + C (30.4%) in *B. paranguensis*, while the complete mitochondrial genome A + T base composition (63.8%) of 12 PCGs was higher than G + C (36.2%) in *B. plicatilis*. The phylogenetic placement of *B. paranguensis* in the genus *Brachionus* with CO1 and Cytb was shown in Figure 1. The rotifer *B. paranguensis* was closely clustered to *B. plicatilis*. However, the positions of some tRNAs (e.g. tRNA-Ile, tRNA-Leu[TTA], tRNA-Phe, and tRNA-Leu[CTC]) of mitochondrial DNA I were different between *B. paranguensis* and *B. plicatilis*. This indicates that the rearrangement of tRNAs is likely occurred in sporadic manner in the genus *Brachionus* (Hwang et al. 2014; Choi, Lee, Lee, et al. 2020).

**Figure 1.**
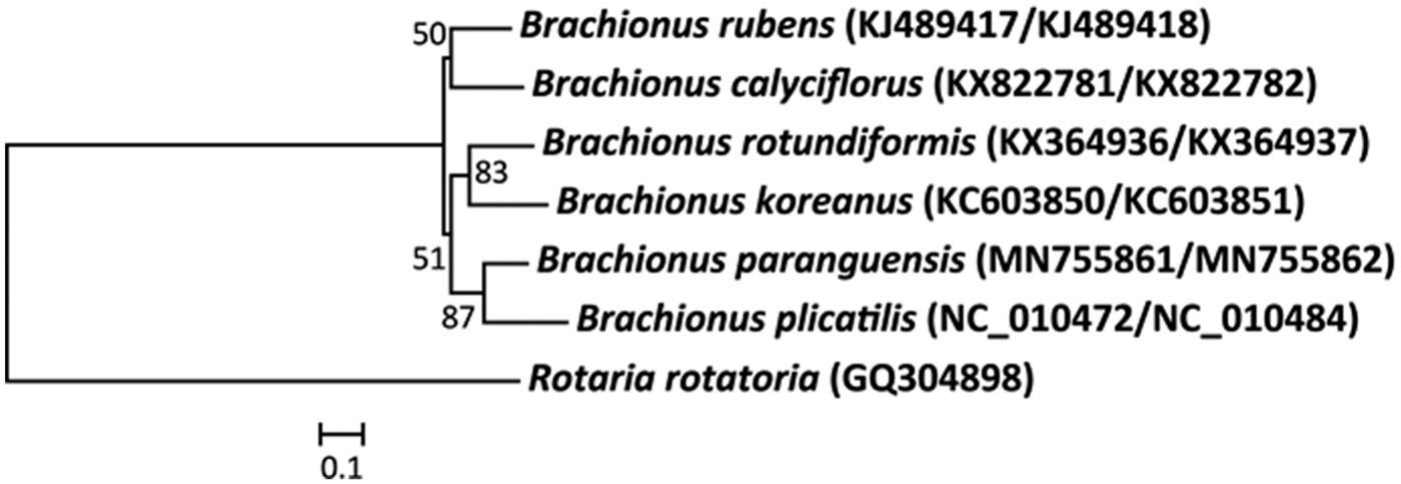
Phylogenetic analysis of the rotifer *Brachionus paranguensis* (‘Nevada’ strain) mitochondrial DNA. We conducted a comparison of seven rotifer species with two mitochondrial DNA genes (CO1 and Cytb) of bdelloid rotifer and *Brachionus* rotifers. The amino acid sequences of seven CO1-Cytb genes were aligned by ClustalW. Maximum likelihood (ML) analysis was performed by Raxml 8.2.4 (http://sco.h-its.org/exelixis/software.html) with GTR + γ + I nucleotide substitution model. The rapid bootstrap analysis was conducted with 1000 replications. The bdelloid rotifer *Rotaria rotiatoria* served as an outgroup. Ln = −12,617.227. Modified from Choi, Lee, Hagiwara, et al. (2019).
